# Case report: Co-occurring autism spectrum disorder (Level One) and obsessive-compulsive disorder in a gender-diverse adolescent

**DOI:** 10.3389/fpsyt.2023.1072645

**Published:** 2023-05-16

**Authors:** Andrea D. Guastello, Corey Lieneman, Brittany Bailey, Melissa Munson, Megan Barthle-Herrera, Miranda Higham, Lindsay Druskin, Cheryl B. McNeil

**Affiliations:** ^1^Florida Exposure and Anxiety Research (FEAR) Laboratory, Department of Psychiatry, University of Florida, Gainesville, FL, United States; ^2^Department of Psychiatry, University of Nebraska Medical Center, Omaha, NE, United States; ^3^Department of School Psychology, University of Florida, Gainesville, FL, United States; ^4^Department of Psychology, West Virginia University, Morgantown, WV, United States

**Keywords:** obsessive-compulsive disorder, autism spectrum disorder, case report, exposure and response prevention, gender identity

## Abstract

This fictionalized case report captures the common themes and considerations during the diagnostic assessment and behavioral treatment of adolescents demonstrating symptoms of autism spectrum disorder (ASD), obsessive-compulsive disorder (OCD), and attention-deficit/hyperactivity disorder (ADHD), as well as gender-diversity concerns. Our patient was a white, non-Hispanic 17-year-old individual who identified as gender-neutral but had been assigned female at birth. Symptoms presented were social withdrawal, rigid rule-following behavior, unusual repetitive behavior, impairments in social communication skills, sensory sensitivity, body dissatisfaction, self-injury, and anxiety related to contamination, perfectionism, and social interactions. These symptoms contributed to functional impairment with school attendance, school achievement, family relationships, and the activities of daily living. This case report summarizes instruments employed for differential diagnosis concerning cognitive functioning, ASD, OCD, ADHD, depression, anxiety, and commonly co-occurring repetitive behavior. This patient was ultimately diagnosed with ASD, level one for both social communication and restricted, repetitive behaviors, without accompanying intellectual or language impairment; OCD with panic attacks; gender dysphoria; major depressive disorder (single episode and moderate); and ADHD. The subsequent 40-session course of cognitive-behavioral therapy with exposure and response prevention (CBT/ERP) to treat OCD tailored to an individual with ASD and gender diversity concerns is described in detail. Components of family involvement are highlighted. As a result, significant improvements in school attendance, OCD symptoms, depression, social relationships, and adaptive functioning were measured. Lastly, recommendations for clinicians are summarized.

## Introduction

The overlapping symptoms of autism spectrum disorder (ASD) and obsessive-compulsive disorder (OCD) are frequently difficult to disentangle. Therefore, differential diagnosis and selection of subsequent treatment are often challenging.

Autism spectrum disorder is a neurodevelopmental condition characterized by significant differences in social communication and restricted/repetitive behavior. Social communication differences must be demonstrated by deficits in social-emotional reciprocity, non-verbal communication, and relationships (1). Restrictive, repetitive behavior must be exemplified by significant differences in at least two of the following domains: repetitive/stereotyped behavior, restrictive behavior, restrictive interests, and sensory sensitivity (1). Restrictive/repetitive behavior in ASD tends to be ego-syntonic, that is, consistent with the individual's self-image, values, and beliefs (2). Approximately 1 in 36 children in the United States are diagnosed with ASD (3).

OCD is characterized by obsessions and/or compulsions. Obsessions are unwanted, intrusive, recurrent thoughts or impulses associated with significant distress. Compulsions are excessive or unrealistic repetitive behaviors reinforced by escape from obsession-related distress (1). Obsessions in OCD are typically ego-dystonic, i.e., inconsistent with an individual's self-image, values, and beliefs (2). In the United States, OCD occurs in about 2.3% of adults (4), and the onset occurs before the age of 15 years in 50% of cases (5).

A meta-analysis indicated that 17.4% of young people with ASD also had symptoms that met the diagnostic criteria for co-occurring OCD (6). Approximately 25% of individuals diagnosed with OCD may also meet the diagnostic criteria for ASD (7). For those diagnosed with co-occurring ASD and OCD, treatment of anxiety, repetitive behavior, and rigid thinking is often further complicated by social and language differences. Relatively few studies have examined effective treatments in the OCD-ASD population (7–13). For a review of this literature and related clinical considerations, refer to Lieneman et al. (14). Nevertheless, it is important that practitioners tailor treatment while considering the unique needs of this population.

## Case description

The following case study is a fictionalized amalgamation of several co-occurring OCD and ASD cases observed in our OCD specialty clinic over the past 5 years. It was fictionalized to highlight common themes and protect patient privacy. All examples pertaining to the assessment battery, psychoeducation, and items for the exposure with response prevention hierarchy are real examples that have been used with many patients in the clinic and are not attributable to any one case. Any data or direct quotations have been fictionalized to reflect common patterns and themes in this patient population.

### Patient information

**Figure 1:**
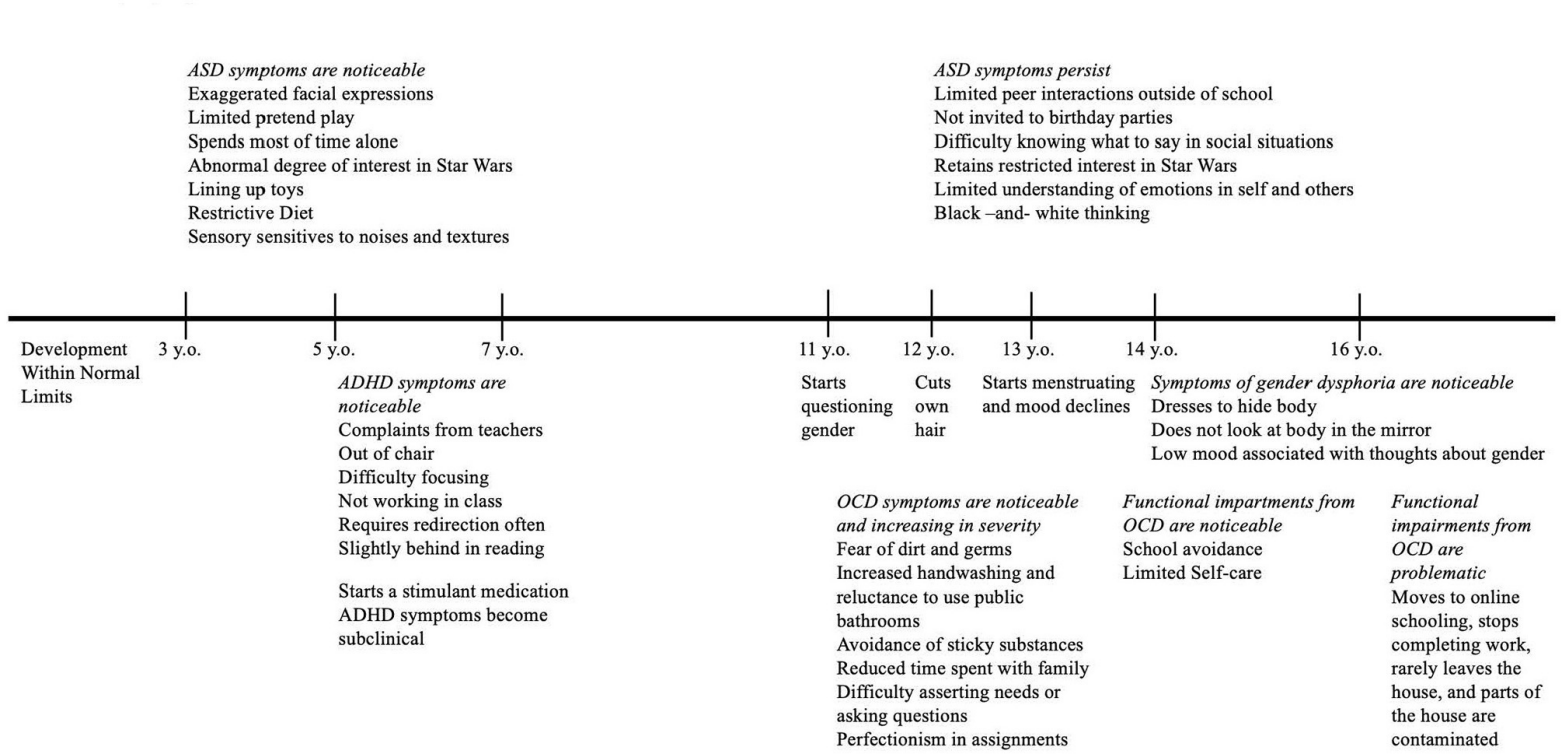
Timeline of symptoms. ADHD, attention-deficit/hyperactivity disorder; ASD, autism spectrum disorder; OCD, obsessive-compulsive disorder.

Alex Doe (fictionalized name) was a white, non-Hispanic, 17-year-old individual (gender-neutral) who was assigned female at birth. Alex identified with they/them pronouns. Alex lived at home with their biological mother, father, and younger brother. They were referred to our OCD specialty clinic by their community therapist who had been treating Alex for 6 months under the diagnosis of adjustment disorder. Alex was referred for assessment and potential treatment of OCD. A timeline of their symptoms from birth until the present day is presented in Figure 1. Family history of psychiatric conditions included ASD for Alex's younger brother and generalized anxiety disorder for their mother.

The family's primary concern was Alex's difficulty completing schoolwork. The family worried that Alex may not graduate high school. School attendance problems dated back 2 years. Alex reported feeling overwhelmed at school due to sensory concerns (e.g., loud noises, sticky substances), contamination concerns, social stresses, previous experiences of being bullied, poor peer interactions, and difficulty using public bathrooms related to contamination and gender dysphoria. School avoidance eventually escalated to school refusal. At the advice of the school district, Alex was approved for homeschooling. Initially, anxiety symptoms decreased, and schoolwork completion increased. However, after several months, symptoms returned and manifested in the home environment, prompting the family to seek community treatment.

At the time of intake into the study, Alex was enrolled in 3 online classes in the 11th grade to repeat incomplete coursework from the previous year. They avoided and expressed anxiety about schoolwork. Most of their time was spent in their bedroom playing video games, reading, and watching Star Wars-related content. Sitting in the same room as family members, preparing and eating meals in the kitchen, and having conversations about schoolwork all prompted significant distress, causing Alex to withdraw from chores and family activities. They also reported concerns about contamination, perfectionism with assignments, and obsessions about being morally good (i.e., scrupulosity). Alex followed strict, self-imposed rules (e.g., “Avoid the lunch table. Re-write. Do not tell lies.”). They also experienced somatic symptoms of anxiety, including panic. Alex withdrew from relationships and interactions with others due to fears of being a “burden” and being “bossy”; they avoided asking questions and making simple requests like asking their mother for lunch money. Alex's mother reported that Alex “thought in black and white,” which exaggerated their fears. She stated that Alex had a limited understanding of social relationships, insufficient nonverbal communication, difficulty understanding the thoughts and feelings of others, sensory sensitivities (e.g., avoiding sticky substances), low distress tolerance, and hitting their head when frustrated. Alex described worrying about rejection and hurting others' feelings. Their mother explained that Alex was diagnosed with attention-deficit/hyperactivity disorder (ADHD) and prescribed a stimulant as a second grader. She noted that ADHD symptoms remained well managed with the medication and denied adverse effects on repetitive or OCD behavior.

The pre-intake packet for our specialty OCD and anxiety disorders clinic includes screening measures for other obsessive-compulsive spectrum disorders (such as body dysmorphic disorder, hoarding, trichotillomania, and excoriation) and conditions that frequently co-occur with OCD. Alex endorsed body dissatisfaction and engaging in other behaviors to lessen this dissatisfaction. A closer examination of Alex's responses indicated that many of their symptoms were related to gender dysphoria rather than body dysmorphic disorder. Specifically, Alex reported distress around the size of their hips and breasts. They explained that they “never really felt like a boy or a girl” and described increasing discomfort with their body since the onset of puberty at age 11. At age 12, Alex had cut their own hair because their parents had been too busy to make them an appointment at a salon. Alex avoided form-fitting clothing. They showered infrequently and in the dark to avoid seeing their body. Alex began menstruating at 13 and reported tearfulness, sadness, social withdrawal, and hopelessness during the week of their period. They attributed these episodes of low mood to being reminded of their sex assigned at birth. Alex's family had named them Alexis and used she/her pronouns for Alex since birth. Alex had been requesting that others call them Alex and use they/them pronouns since age 13. This caused family conflict, but the family eventually complied. The family still avoided extended family get-togethers due to discomfort regarding “imposing” these expectations on others (e.g., grandparents). Alex's mother described Alex's assertion of their identity as “obsessive.” Alex reported feeling certain about their gender identity and denied gender-related checking (e.g., asking others how they can tell what gender they are, seeking reassurance that their identity is accurate, engaging in traditionally gendered behavior, and judging their internal response). Please see Figure 1 for a timeline of Alex's symptoms since early childhood.

## Diagnostic assessment

Based on the intake assessment packet and clinical interview data, ASD and OCD were suspected. The treatment team believed diagnostic clarity would allow for a more focused and streamlined approach to treatment; therefore, the following recommendations were shared with the family:

(Transcript of the interaction between the therapist, Alex, and their mother).

*Therapist:* I want to let you know that you are in the right place. We are confident we can help you. Based on what I heard in this interview today, I noticed anxiety that could be OCD. I also noted some difficulties with social interactions with adults and people your age that have been going on for a long time. Does that sound right?
*Alex:* Maybe.
*Alex's mother:* Yes, that sounds right.
*Therapist:* It sounds like we are on the same page. Before we begin anxiety/OCD treatment, it would help me if I knew more about how your brain works. Psychologists are getting better at noticing when young people assigned female at birth have brain differences or “neurodevelopmental disorders.” When you were young, your pediatrician did a great job noticing your ADHD, but we didn't know as much about other neurodevelopmental differences back then. Based on your history, I wonder if you might have something called autism spectrum disorder or “ASD.” Have you ever heard of that?
*Alex:* Yeah, some kids at school have that, but they can't really talk or anything. Are you saying that I have that?
*Therapist:* Not exactly. Autism can look different for different people. The amount that symptoms get in the way in life varies; that's why it is now called the autism spectrum. If someone can't use words without other communication tools, their symptoms are impactful. While you are not as impacted as your classmate, your symptoms might still fall somewhere on this spectrum.
*Alex's mother:* Is this really necessary? How would this help us?
*Therapist:* I cannot require you to get this evaluation, but I highly recommend it. Regardless of the final diagnosis, the evaluation results will influence how we go about therapy and could impact how Alex approaches future life decisions. Like the old idiom says, “Measure twice. Cut once.” I think that spending time on the evaluation now may save time and resources in the long run.

Alex and their mother agreed to the comprehensive ASD evaluation. The relevant results and findings are presented below. Of note, the majority of the scales used below have combined gender norms. The Social Responsiveness Scale (SRS) was the only measure that had gendered norms. For Alex, we used female norms because female norms are more sensitive to social impairments and because they best represent the social expectations that Alex is facing in most social environments currently. For other patients, it may be appropriate to report and interpret findings using both sets of norms.

### Cognitive functioning

#### The Wechsler Adult Intelligence Scale, Fourth Edition (WAIS-IV)

The WAIS-IV (15) is an individually administered, norm-referenced test that evaluates the intellectual functioning of individuals compared to their same-aged peers.

Alex's scores on the WAIS-IV indicated cognitive abilities in the high average range, with relative strengths in Verbal Comprehension and Working Memory. Alex's Perceptual Reasoning score fell in the average range, and their Processing Speed score was low average.

### ASD-related testing

#### Autism Diagnostic Observation Schedule–Second Edition (ADOS-2), module 4

The ADOS-2 (16) is part of the gold-standard evaluation for ASD. It is comprised of standard activities that allow the examiner to observe behaviors, including developmentally appropriate communication and socialization that have been identified as important to the diagnosis of ASD (17, 18).

During the ADOS-2, Alex's spontaneous speech consisted of several complex and grammatically correct sentences with a normal rate and tone with no immediate echolalia or stereotyped speech. Alex briefly looked at the examiner while the examiner spoke, but eye contact was not maintained. Alex offered information about their experiences on previous trips and asked the examiner about her experiences. They also integrated vocalizations with gestures when speaking. Alex displayed shared enjoyment with the examiner throughout the administration, as evidenced by shared laughter. In response to interview questions, Alex labeled others' emotions and described internal experiences associated with emotions. They had limited insight into relationships and their role in relationships. Alex described having to touch someone else as a difficult part of marriage. During ADOS administration, Alex mentioned knowing “I am a biological girl” because “I have a vagina, but I do not feel like a boy or a girl.” Alex did not engage in any sensory-seeking, sensory-avoiding, obviously repetitive, or ritualistic behavior, other than fidgeting with their hair. Based on the observations during the ADOS-2, Alex's symptoms fell in the “non-spectrum” range.

#### The Autism Diagnostic Interview-Revised (ADI-R)

The ADI-R (19) is a standardized, semi-structured interview used to assess developmental history in the domains of (1) Communication, (2) Reciprocal Social Interaction, and (3) Restricted, Repetitive, and Stereotyped Patterns of Behavior. The ADI-R also assesses for the presence of symptoms at or before 36 months of age. Scores derived from this interview using a diagnostic algorithm are compared with cutoff scores indicating the likelihood of ASD.

Alex's mother was interviewed using the ADI-R. She reported developmental concerns for Alex related to social interaction. When recalling Alex's behavior between the ages of four and five, Alex's mother reported that Alex made exaggerated facial expressions in family photos, had difficulty engaging in pretend play and responding appropriately to social situations, and spent most of their time alone. She stated that Alex used verbal communication to get attention and engage in reciprocal conversation, but much of this conversation related to Star Wars. Alex rarely integrated verbal and nonverbal communication skills (e.g., eye contact and gestures). As a child, Alex's speech included idiosyncrasies; for example, they referred to people based on traits rather than their names. Regarding restrictive, repetitive, and stereotyped behavior, Alex's mother reported that when they were younger, they were preoccupied with Star Wars, frequently lined up toys, often rocked their body, and avoided foods with slimy textures. Alex's mother stated that, before 36 months of age, Alex had tantrums, but no other concerns were reported. Based on this information, Alex's scores exceeded the cutoff in two domains (Reciprocal Social Interaction and Restricted, Repetitive, and Stereotyped Patterns of Behavior) and met the cutoff in the Communication domain. However, Alex's score was below the cutoff for Abnormality of Development Evident at or before 36 months.

### Parent-report measures for ASD

#### Social Communication Questionnaire (SCQ)

The SCQ (20) measures the effective use of social communication, which may be impacted by ASD. Alex's mother's report resulted in a score that directly met the cutoff, suggesting some social communication difficulties, but these difficulties may not have been clinically significant.

#### Social responsiveness scale (SRS)

The SRS (21) is a questionnaire that evaluates the presence of social impairment as it relates to ASD. Alex's mother's report indicated that Alex has some difficulty with social responsiveness overall, as indicated by a total score directly at the cutoff. More specifically, Alex's mother's report indicated significant difficulty with social motivation and social cognition, with limited engagement in restricted repetitive behaviors. Scores for social awareness and social communication directly met the cutoff, indicating that Alex had difficulties with these two aspects of social interaction, but they may not have been clinically significant.

#### Adaptive Behavior Assessment 3rd Edition (ABAS-3)

The ABAS-3 (22) is a questionnaire that assesses one's adaptive skills across their lifespan, which is imperative when evaluating for ASD. Alex's mother's report indicated that Alex's overall adaptive behavior was “low,” indicating Alex's abilities to adequately communicate, socialize with others, and independently engage in practical life tasks were “low.” Additionally, their ability to engage in functional academics was “very low.”

### OCD and related testing

#### Children's Yale-Brown Obsessive-Compulsive Scale (CY-BOCS)

The CY-BOCS (23) is a semi-structured interview used to assess the severity of obsessions and compulsions over the previous week. The children's scale was used with Alex because the items in this scale relate more to school settings, as opposed to the workplace. Alex and their mother were interviewed together. Alex's score indicated that they experienced “severe” obsessive and compulsive symptoms.

#### Family Accommodation Scale (FAS)

The FAS (24) for OCD is a caregiver-report measure that evaluates the types of accommodations caregivers make for their child's obsessions and compulsions. It can be helpful for treatment planning. Alex's mother's report indicated that she made significant accommodations (reassurance, avoidance of certain topics, etc.).

#### Rating scales for commonly co-occurring disorders

Several questionnaires were administered to assess for commonly co-occurring disorders with OCD, including depression, anxiety disorders, and tic and Tourette's disorders.

#### Beck Depression Inventory, Second Edition (BDI-2)

The BDI-2 (25) is a self-report questionnaire designed to evaluate the severity of depressive symptoms. Alex's scores indicated that they were experiencing moderate symptoms of depression.

#### Screen for Child Anxiety Related Disorders (SCARED)

The SCARED (26) is a screener for childhood anxiety disorders, including panic disorder, generalized anxiety disorder, separation anxiety, social anxiety, and school avoidance. Alex's scores were above the clinical cutoffs on the scales of School Avoidance, Panic, and Generalized Anxiety.

#### Yale Global Tic Severity Scale (YGTSS)

The YGTSS (27) is a clinician-rated instrument that evaluates the presence, frequency, intensity, complexity, interference, and impairment of symptoms related to tic and Tourette's disorders in children and adolescents. Scores indicated that Alex was not experiencing any motor or vocal tics.

#### Repetitive Body Focused Behavior Scale–parent (RBFBS)

The RBFBS is a rating scale that evaluates caregivers' perceptions of their child's body-focused repetitive behavior, such as hair-pulling, nail-biting, or skin-picking. Alex's mother's ratings on this scale fell within normal limits, indicating that Alex was within normal limits of nail-biting and did not report any hair-pulling or skin-picking.

#### Conners, Third Edition–parent form (Conners 3)

The Conners 3 (28) is a rating scale that obtains information concerning parents' perceptions of their child's behavior related to ADHD and concurrent disorders. Alex's mother's report indicated that Alex engaged in behavior related to ADHD (e.g., inattention, hyperactivity/impulsivity, executive functioning difficulties, learning problems, and defiance), but not to a clinically significant degree.

### Clinical findings

Based on the results of our clinical interview and observations, structured interviews, and self- and parent-reports, Alex's symptoms met the Diagnostic and Statistical Manual of Mental Disorders-5-Text Revision (DSM-V-TR) (1) criteria for the following disorders: ASD, level one for both social communication and restricted, repetitive behaviors, without accompanying intellectual impairment or language impairment; OCD with panic attacks; gender dysphoria; major depressive disorder, single episode, moderate; and ADHD, combined presentation (by history).

In addition to ego-syntonic restrictive and repetitive behavior related to autism (sensory avoidance, fascination with Star Wars, repetitive, self-stimulatory movements, etc.), Alex demonstrated clear evidence of obsessions around schoolwork, contamination, perfectionism, not hurting others' feelings, as well as compulsions that included avoidance and re-writing. These reports were consistent with their report on the CY-BOCS, which indicated that Alex experienced severe obsessive and compulsive symptoms. It was clear that these ego-dystonic worries and behaviors were separate from those of ritualized and repetitive behaviors associated with autism, as they caused significant distress. Thus, both ASD and OCD were diagnosed.

Alex reported that they had never connected with binary gender identities, and they began to experience distress related to inconsistency between their body and their gender identity when they began puberty. Family impairment had increased since Alex began to request to be treated in a gender-neutral way. Alex described high levels of dissatisfaction related to the shape of their body and the experience of menstruation. As such, Alex's experiences were consistent with a diagnosis of gender dysphoria.

Finally, Alex was diagnosed with ADHD in the second grade and had been prescribed stimulant medication ever since. Our evaluation showed symptoms consistent with ADHD including a relative weakness in processing speed and inattentive and hyperactive symptom scores in the at-risk ranges. Therefore, the diagnosis of ADHD, combined presentation, was retained.

### Testing feedback

We included all family members Alex chose, even if they would not be involved in treatment.

### Referrals

**Figure 2:**
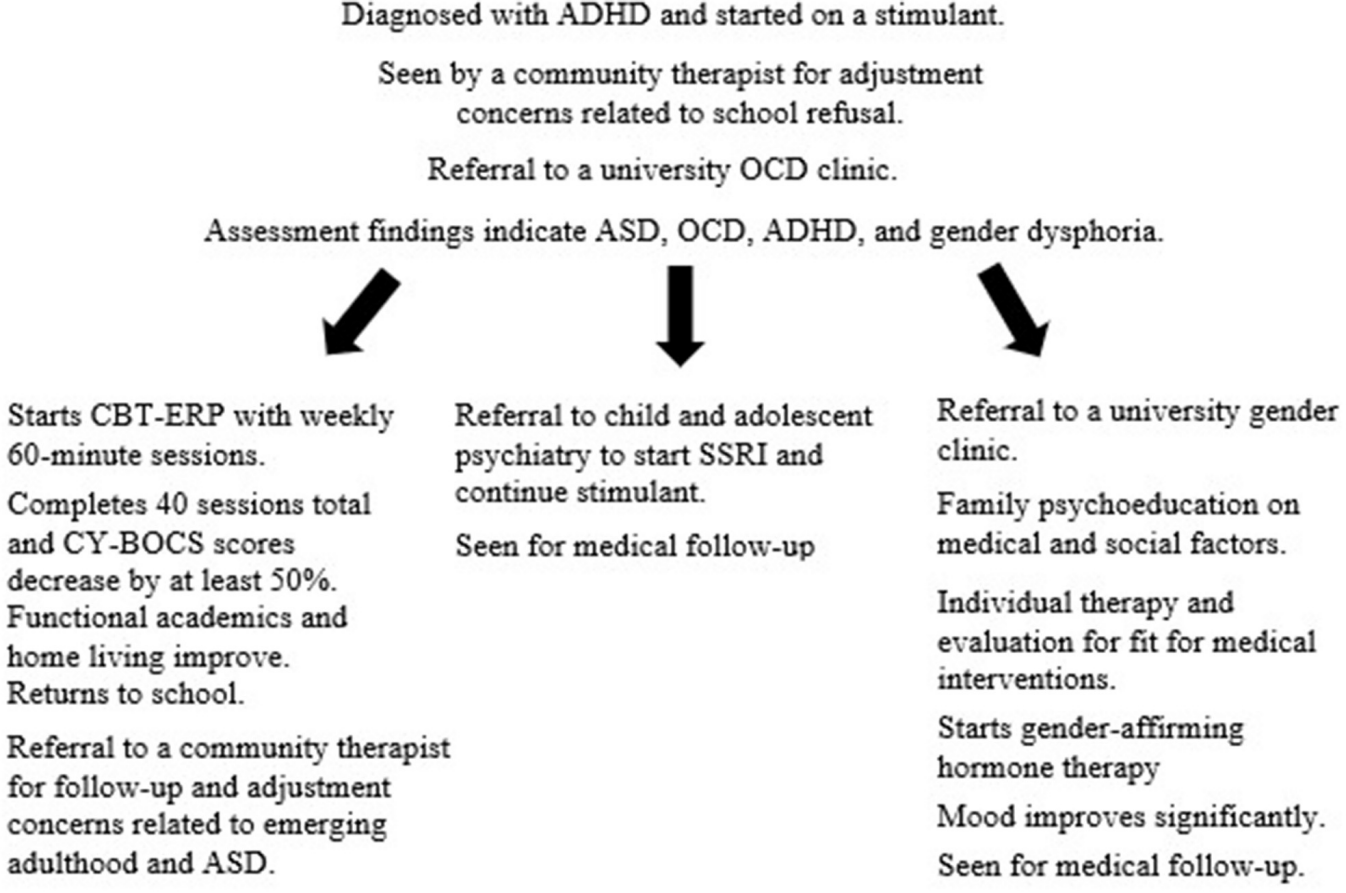
Sequence of treatment. ADHD, attention-deficit/hyperactivity disorder; ASD, autism spectrum disorder; CBT-ERP, cognitive-behavioral therapy with exposure and response prevention; OCD, obsessive-compulsive disorder; SSRI, selective serotonin reuptake inhibitor; CY-BOCS, Children's Yale-Brown Obsessive-Compulsive Scale.

In addition to the primary intervention described in detail below, two referrals were made to run concurrently with Cognitive Behavioral Therapy with Exposure and Response Prevention (CBT-ERP): (1) to a multidisciplinary gender clinic for brief family education and ongoing medication management including gender affirming hormone treatment if deemed appropriate and (2) to child and adolescent psychiatry for medication management of ADHD and possibly OCD. Please see Figure 2 for a summary of the services and referrals Alex received.

(What follows is a transcript of the primary intervention between the therapist, Alex, and their family).

#### Sex differences in ASD

Therapist (to Alex): I know you must be frustrated that it took so long for someone to recognize that you have ASD. Individuals with ASD assigned female at birth are more often diagnosed later than those assigned male at birth or not at all (29). This results from in-born abilities and societal pressures for those assigned female at birth to excel at social and communication skills. Therefore, those assigned female are often better at *masking* their symptoms (30, 31).

#### ASD vs. ADHD

Therapist (to Alex): Many people with ASD also have ADHD. ADHD impacts our *executive skills*: our ability to plan, organize, monitor, and control our minds and bodies. This is often noticed in schoolwork as it takes lots of attention for boring tasks and requires you to sit still and quiet for most of the day. ADHD can also impact our relationships. It can be hard to keep friends when we say things impulsively that can hurt other people's feelings, get in other people's personal space, or can't focus on what they say. However, the number of social difficulties you are having is above and beyond what we see in ADHD alone. It's not just that you have difficulty controlling your mind and body in social situations, but understanding relationships is challenging, and even when your medication is helping you control your mind and body, you are often unsure of what to say or do. Finally, people with ADHD do often report *hyperfocus* where they can focus on one interesting task for hours on end. This can be confused with a restricted interest. The difference is that, in ADHD, the task can vary from video games one day to a cool art project or model airplane the next. In ASD, there is much less variation in the interest [sic].

#### ASD vs. OCD

Therapist (to Alex): We've talked about ASD and restricted behaviors. Now, let's think about how these symptoms can overlap with OCD. In OCD, an obsession is a recurring thought, image, or impulse that causes distress. This is key; obsessions are distressing. Sometimes in our daily life, we say someone is “obsessed” with something to mean that they “like it a lot.” Some people have said you are “obsessed” with Star Wars, but Star Wars doesn't make you upset. It makes you happy. To be more clinically accurate, we can say you have a restricted interest in Star Wars. However, you do have some obsessions, like your fears about hurting others and germs. You also have some sensory sensitivities that overlap with this idea of things being *just right*. You spend a lot of time and energy trying to make things feel *right*, and when they feel *wrong*, you get upset.

When someone has obsessions, they usually also have compulsions. A compulsion means doing something to make yourself feel better when your obsessions are bothering you. Over time, these things take up too much time or don't make sense anymore. Some of your compulsions are washing your hands and avoiding making simple requests. You don't like these things, and they get in the way of your life. Compulsions are different from some of your ASD symptoms like following routines because compulsions are related to your obsessions. We call this obsessive-compulsive disorder or OCD. It may be upsetting or overwhelming to hear about all these labels. The good news is that there are excellent treatments for OCD. Now that we know your diagnosis, we can do some things to help.

#### Gender identity concerns vs. OCD

Therapist (to Alex): As a reminder, *obsessions* are uncomfortable, intrusive thoughts related to uncertainty or doubt. People with OCD feel compelled to do something to make that thought go away. For instance, Alex may think, “What if I get sick?” and wash their hands to make that thought go away. However, Alex doesn't have uncertainty or doubt about their gender. They know who they are, and they have known for a long time. Alex's thoughts about being non-binary don't feel uncomfortable unless they are about the effort it takes to be comfortable in their body or [sic] other people's negative reactions. Those are the things that Alex needs support around.

Our healthcare center has a gender clinic for kids and teens, and they specialize in helping people like Alex make the changes they need to feel good about themselves. Our patients tell us that the clinic does a good job of providing more education to families about gender issues. They have medical providers who can provide education about hormones and answer your questions if you are interested.

However, the biggest piece of helping Alex right now isn't medical, it's social. We need to focus on changing the way people respond to them. You don't have control over everyone in Alex's life, but that's okay because you matter the most. Your support and the support of the rest of Alex's family are crucial. Research shows that trans and gender-diverse individuals like Alex are at risk [sic] serious problems like suicide and homelessness. The single most powerful way to prevent those things is by hearing them and respecting them when they tell you who they are.

#### Medication for OCD

Therapist (to Alex): Alex, there is one more thing we can do to help your OCD. Research has shown that a combination of medication and exposure and response prevention has the greatest impact on OCD. Many of my patients find that the medication turns down the volume of their obsessions. They say that, while they might still have some obsessions, they feel less frequent and when they are there, they are easier to dismiss, more like background noise. Some patients also say that the medication can help make their body calm down a bit. It makes it so when they are anxious or panicky, it isn't as overwhelming. Because it seems like OCD is impacting your life greatly right now, I wonder if you and your family would want a consultation with our child and adolescent psychiatrists. I know your pediatrician has been prescribing your ADHD medications, but when we have a couple of things going on like OCD, ASD, and ADHD, it can be helpful to have a specialist. Our child and adolescent psychiatrists can answer any questions you have about medications and start you on medication if you are interested. They are good at not being pushy. I heard you say that you aren't interested in another medication, since you are already taking something for your ADHD. I wonder if they could be a good resource so you could at least learn more about them, so you have all the information when making your decision.

## Therapeutic intervention for OCD

Below are the session-by-session outlines that illustrate modifications of ERP for adolescents with co-occurring OCD and ASD. While there are several unique elements to our approach, the foundation of these outlines is the well-established ERP protocols of Abramowitz (32, 33), Franklin and Foa (34), Himle and Franklin (35), and Kircanski and Peris (36). Although these sources vary slightly, the general session structure is largely consistent.

### Session one

We started the session with the expectation that the first session would be mostly psychoeducation concerning the brain-body connection in OCD and how to retrain the body. Whenever possible, we used examples directly related to the patient's experience. We drew out the following graphs on whiteboards to illustrate.

#### Psychoeducation on OCD in the context of ASD

(Transcript of therapist's conversation with Alex and their family).

**Figure 3:**
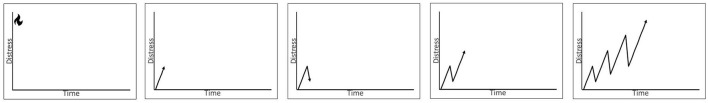
Illustration for psychoeducation on behavioral reinforcement of obsessions and compulsions.

Therapist (To Alex): We all have an alarm system in our bodies called the fight-flight-freeze system that is important for keeping us safe. Without it, we would do risky things like walk into traffic, walk too close to large cliffs, or forget to get dressed before going out in public. For instance, if I smelled smoke in the office right now, my alarm system would turn on (Therapist draws out Figure 3). Almost immediately, I would feel distressed in both my body and my mind. My body would move blood from my digestion into my arms and legs, my breathing would increase, so I have more oxygen in my body, my heart would beat faster so that oxygen can get to my muscles, my pupils would dilate so I could see better, and my head might feel funny as my brain tries to make quick decisions. All of this keeps me safe so that I can run away from the danger or maybe even break a window if I need to get out. You told me in the evaluation that when you feel anxious or panicky, your heart beats fast, your breathing changes and you feel like you can't breathe, and your brain gets fuzzy. That is your fight-flight-freeze system and even though it's scary, this is a natural process in your body.

However, with OCD and panic, this alarm system gets hijacked, so you get lots of false alarms. One example you gave me is a fear of germs in the bathroom. Most people wash their hands after going to the bathroom. However, your brain gets extra worried about the bathroom germs. The first time you thought hard about the germs, you washed your hands and you felt mostly better afterward (Therapist begins to draw Figure 3). Usually, you don't feel all the way better because there is still some doubt; “Did I really get all of the germs?” The next time you used the bathroom you had the same thought; when someone has OCD, we call these thoughts *obsessions*. You decided to make yourself feel better by washing your hands just a touch longer or using just a little more soap. Just like last time, you felt mostly better, but there is always a little bit of doubt, “Did I really get all of the germs?”. We keep having the same patterns.

**Figure 4:**
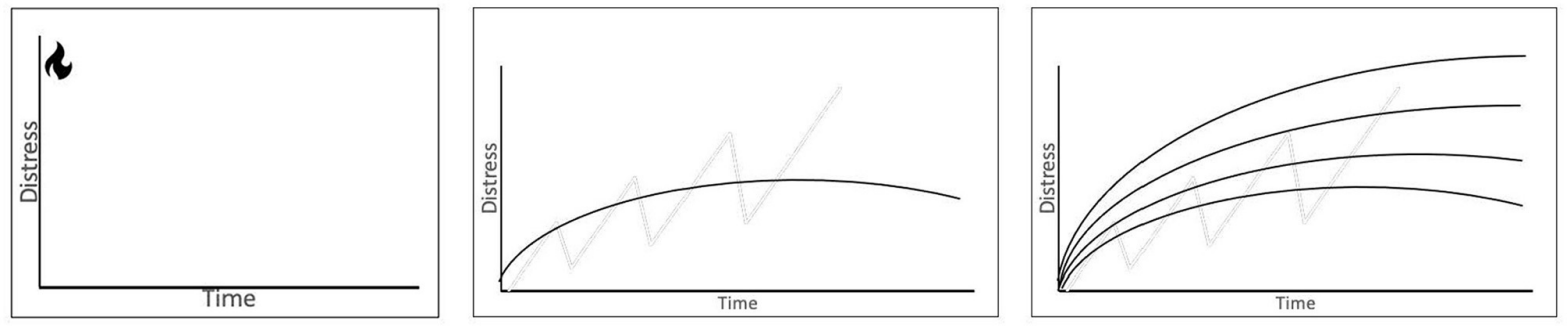
Illustration for psychoeducation exposure therapy, distress tolerance, and between session habituation.

*Therapist:* What happens the next time you use the bathroom?
*Alex:* I think my hands are dirty and I wash them.
*Therapist:* Do you wash them the same amount or more?
*Alex:* Well, probably more.
*Therapist:* Do you feel better?
*Alex:* Well, I feel good for a minute but then I worry again.
*Therapist:* Exactly. These things you do to *feel better* are called compulsions, and over time they make our lives harder. Over months and months, this obsession gets stronger and stronger. Your ability to listen to your obsessions and not do compulsions decreases and you start avoiding things to try and stop your obsessions. First, you stop using public bathrooms. Then you stop using other bathrooms. Over time, you only use your one bathroom and no one else can go in it. Did all that stop your obsessions or make you feel better?
*Alex:* No, I still think about germs a lot.
*Therapist:* Exactly, you still think about germs a lot, and your world ended up shrinking. It used to include public places and [sic] school. Then it only included school and home. Then it only included home. Now it only includes your bedroom, bathroom, and sometimes the kitchen. Do you like things this way?
*Alex:* No, not really.
*Therapist:* Would you like to have some more of your world back?
*Alex:* Sure, but how?
*Therapist:* Well, over years and years of research, the best therapy we can offer to help with OCD is this thing called exposure with response prevention—we call it “ERP.” The whole idea behind ERP is that we take this big scary thing, like using public bathrooms, and we break it into smaller parts. Maybe, we start by touching the door to the bathroom or standing in the bathroom but not intentionally touching anything yet. You *approach* the situation, which does end up triggering some of your obsessions and then you resist the urge to do a compulsion. In your case, this would mean you would not wash your hands, and you would stay in the bathroom for a preset amount of time (maybe 15 min), so you are not avoiding or escaping the situation nor are we doing any compulsions. In ERP, we call these activities “exposures,” but I like to call them *practice*. When you practice being in uncomfortable or even scary situations like this, your body will eventually adjust, and your world can start expanding again.
*Therapist:* I'm going to be honest with you. The fact that you have ASD means this process will probably be a bit more difficult for you than it is for some other people. When a person with ASD gets upset, it can take a long time for their body to calm back down. If we look back at my graphs here, this is what is going to happen (Therapist starts drawing Figure 4). We are going to start with a challenge like touching the bathroom door, and your anxiety or general distress is going to increase. Hopefully, we will pick reasonable enough challenges that it won't increase too much. When this is happening, your mind is going to say things like, “This is too hard,” “I can't do this,” “I'm going to freak out,” or “Why is this woman asking me to do this?” But, if you can wait out this first part, eventually, your distress will level off. It won't keep going up and up. If you can stay in the situation, you also learn, “I can handle this,” or “I can be uncomfortable without freaking out.”
*Therapist:* Next time we meet, we are going to build this thing called a hierarchy where we take a big task like “using a public bathroom” and break it into smaller parts that get a little harder as we go. For example, after touching the door, we might touch the door [sic] then touch the toilet flusher for 5 min. We could sit on the floor and play two rounds of UNO. We could sit on the toilet with our pants on, so our pants get all germy. What we put on the hierarchy will be based on exactly what your OCD obsesses about and how hard you think a practice activity will be.
*Therapist:* Because you have autism, your body will need extra practice before it starts to learn that it doesn't need its fight-flight-freeze system in the bathroom. We are going to have to practice. We are going to practice in the same bathrooms, but also in different bathrooms so that your body can learn that you can handle it in all types of bathrooms. If you agree to try this whole ERP thing, we are going to take this approach for several types of obsessions and general discomforts you have including sticky things, cooking, talking to others, and assertiveness. What do you think?
*Alex's mother*: Well, I did some reading online after we were first told that they might have OCD. People were saying that ERP is traumatizing, and this all seems scary. Is this traumatizing?
*Therapist:* I'm so glad you asked me that question. It is important that we all agree on this treatment before we start. There are always some people who have had a bad experience with therapy or where the therapist and patient match was not good. I can't speak directly to what you saw online because I didn't see exactly what you were looking at. But, when people have found ERP too challenging, it is usually because the exposures or practice moved to the more difficult tasks too quickly or because there was not enough repetition to allow for someone's body to learn to calm down across several days of trying a new task.
*Therapist:* I want you to feel that you can trust me during this process. Trust doesn't mean that you always like me or that you never doubt that this is a good idea, because OCD loves to find things to doubt, but that [sic] when you zoom out and think about things, you realize that you need some help re-expanding your world. There are three things I will promise as your exposure therapist: (1) I will never ask you to do anything that I am unwilling to do myself, (2) I will never surprise you with a practice exercise we had not discussed before, and (3) [sic] being annoyed or tired of me at some part of this process is normal, and we will be able to work through that if you choose to.
*Alex's mother:* Well, that sounds good, but they are just so anxious all the time. I don't want to do anything to make it worse.
*Therapist:* I hear you, and it sounds like you are a wonderful mother trying to protect your baby. That's a natural thing to do. However, Alex is already incredibly anxious. Unfortunately, for a young person with OCD and ASD, we have to do things a little bit differently. The good news is that with your strong parenting instincts and my guidance, we can help Alex to make some life-changing progress. What do you think Alex?
*Alex:* Mom, I know I'm not happy living like this.
*Therapist:* Having OCD is challenging and confusing, especially when you also have autism. I hope you're willing, even if you are scared, to try something different.
*Alex:* I want to try.
*Therapist:* Mom?
*Alex's mother:* I guess you're right. Things aren't working. I'm just scared of making it worse. Alex, if you want to try this, I will try too.
*Therapist:* Well wonderful. I'm excited to hear that you are ready to try something new, even if it is scary. Alex, the next session will be our hierarchy-building session. This session will be hard because I will have questions for you about your anxieties, but remember I am not going to surprise you with anything. I'm confident we'll be able to make a great hierarchy. Mom, having you present in sessions with Alex is going to be essential for you to learn how to support them.

### Session two: hierarchy-building

For this session of a little over 1 hour, we developed and followed a session outline detailed below. The approach and protocol to be adopted in the session have also been discussed.

#### Session outline

1. Up to 10 min: Ask for thoughts or reactions from the last session. Review graphs from the last session and prompt for as much teach-back from the patient and family as possible.
2. Up to 30 min: Try and get items for each domain of OCD the patient is experiencing. At a minimum, find an easy, medium, and hard task for each domain.
3. Up to 5 min: Discuss high-value items that can be used as rewards for completing exposure tasks in session and as homework.
4. Last 15–20 min: Try a first exposure. Pick an easy exposure and set the time limit to allow time for brief processing of the exposure before the session ends.
5. Last 5 min: Ask about what the patient learned about their anxiety, about their body, or about their brain, from doing the exposure. Congratulate them and set an expectation that the next session will include more psychoeducation for the family and will increase the time spent in an exposure. A brief reminder that exposure tasks will be slow and thoughtful is usually helpful as most patients are stuck thinking about the hard tasks that were discussed earlier in the session.

### Building the OCD/ASD hierarchy

#### The role of subjective units of distress ratings (SUDS)

With ASD cases, the exposure process is typically more focused on distress tolerance than habituation [see Lieneman et al. (13), for more information]. Because we are *not* expecting within-session habituation on tasks, we are not as focused on SUDS ratings to determine the end of an exposure task. Instead, we are setting operationalized goals based on observable behavior at the start of every exposure. Therefore, we do not need exact SUDS ratings as estimates of “easy,” “medium,” or “hard” usually provide enough information and are easier for the patient to generate. We will still attempt to grade the tasks, by starting with easy or medium tasks, as starting with more difficult tasks can easily become overwhelming for individuals with ASD, decreasing buy-in and consumer satisfaction and increasing our risk of drop-out.

#### Types of tasks to include

It is always preferable to include exposure tasks that have a real-world functional component. Since individuals with ASD take repeated attempts before experiencing any habituation or sense of mastery of a task, it becomes even more important that the chosen tasks align with the actual issues they are facing in their life currently. When creating the hierarchy or choosing the exposure of the day, always align the task with the current functional limitation. Please see Table 1 for an example hierarchy for Alex.

**Table 1:** Example hierarchy for Alex.

**Domain of OCD**	**Task**	**Estimated difficulty**
“Just Right”/Perfectionism	Coloring a picture with “wrong” colors and outside of the lines.	Easy
“Just Right”/Perfectionism	Sending a text message with a typo in it.	Medium
“Just Right”/Perfectionism	Turning in a homework assignment with a known error.	Hard
“Just Right”/Sensory Sensitivity	Sticky items on hands. Washing allowed after 20 minutes. Stimuli included: syrup, jam, mushed gummy candies, mayonnaise.	Easy
“Just Right”/Sensory Sensitivity	Washing dishes, using sponge and dish soap. No washing hands afterward.	Easy
“Just Right”/Sensory Sensitivity	Touching something sticky then touching face. Washing allowed after 15 minutes.	Medium
“Just Right”/Sensory Sensitivity	Touching deli meats. No washing afterward.	Medium
Harm	Holding a knife: stimuli including plastic knife and butter knife.	Easy
Harm	Holding a knife: stimuli including a butcher knife and tomato knife.	Medium
Harm	Using the stove to fry an egg.	Medium
Harm & “Just Right”/Sensory Sensitivity	Cutting fruit to make a fruit salad.	Hard
Harm & “Just Right”/Sensory Sensitivity	Making a breakfast sandwich with toasted English Muffin, fried egg, cheese, and deli ham	Hard
Contamination	Touching bathroom door handles. Washing allowed after 45 minutes and touching multiple handles.	Easy
Contamination	Touching flusher to the toilet. Washing allowed after 15 minutes.	Easy
Contamination	Playing UNO on bathroom floor (of single toilet bathroom). No washing.	Medium
Contamination	Using a bathroom in the office. Washing hands allowed per CDC guidelines.	Hard
Contamination	Cleaning out their lizard's terrarium.	Hard
Social Anxiety/Harm	Complimenting something in someone's office.	Easy
Social Anxiety/Harm	Complementing something on someone's body like clothing or hair.	Easy
Social Anxiety/Harm	Make a request of someone: “Can you get me a glass of water?”.	Medium
Social Anxiety/Harm	Making a demand of someone: “Get me a glass of water.”	Hard
Social Anxiety/Harm	Asking a question in class.	Hard
Social Anxiety/Harm	Asking the teacher a question about an assignment via email.	Hard
Social Anxiety/Harm	Gently correcting someone using the wrong pronouns.	Hard

#### The need for rewards

Many caregivers can get stuck on the idea that “My child should be doing this anyway. Why should they get a reward?”. Remind them that exposure therapy is difficult and we all can benefit from additional support when we are trying new and difficult things. Common rewards could include a preferred snack after sessions, extra screen time, later bedtimes on the weekends, picking dinner on Friday night, and earning points toward a new game. How big the reward is and how to space them out over the week is no different from other behavioral management techniques. For patients that engage in avoidance and escape, it can be helpful to structure sessions so that if all the practice tasks are completed quickly, the patient can leave the session early.

### Session three: first exposure session

This session is a combination of exposure tasks that will continue throughout treatment and additional psychoeducation for caregivers or loved ones. For children, this is almost always a parent or guardian. For adults, this can either be a parent or a spouse depending on age and current life circumstances. We often refer to this loved one as an “ERP Coach.”

### Session outline

1. Up to 10 min: Ask for thoughts or reactions from the last session and identify a current stressor. Set the expectation that the current session will continue psychoeducation and increase time spent on practice.
2. Up to 20 min: Discuss with family members the difference between giving reassurance and providing encouragement. Discuss how to use selective attention to reduce the attention given to OCD and reinforce approach behaviors (Handouts, one of which is adapted from the Parent Child Interaction Therapy Manual, have been provided in the Supplementary material) (37).
3. Up to 20 min: Pick a low-level practice task from the hierarchy. Set a goal to stay on that task for around 20 minutes. Ask questions focused on the patient's sensory experience to build mindfulness skills. Prompt the caregiver to use praise approach behaviors as has been taught earlier in the session.
4. Last 5 min: Ask about what the patient learned about their anxiety, about their body, or about their brain, from doing the exposure. Congratulate the patient and their family for completing a longer exposure.

### Example of psychoeducation for family members

*Therapist:* Before we get started, I want to talk about the difference between reassurance, accommodation, and encouragement. As we go through our hierarchy, Alex is going to need some encouragement. This is normal because this stuff is hard. There are lots of ways that family members accidentally end up accommodating OCD. It is important for us to try and reduce our accommodation because if we keep accommodating, OCD gets to keep a hold on us. Alex's mother, what are some common things you say to Alex when they are upset?
*Alex's mother*: Well, I try and make them feel better. I say stuff that every mother says like, “Don't worry.”, or “It will be ok.”
*Therapist*: You're right, we all say stuff like that when we see someone we love hurting. Does it work for Alex? Do they feel better? Alex, do you feel better?
*Alex*: Sometimes I feel better, but it doesn't last too long.
*Alex's mother*: Yeah. They usually end up asking me questions again, or I say the same thing over and over.
*Therapist*: Exactly. We end up in a similar pattern as our other compulsions, right?
*Alex's mother*: I guess that's true.
*Alex nods slightly*.
*Therapist:* What we need to do is find ways to be encouraging without getting into an argument with OCD. If I try to reassure by saying, “It's going to be ok.” OCD wants to argue. It wants to tell you all the reasons it won't be ok. If I say, “You're doing great working through this,” I may not completely believe you, but it will be much harder for OCD to argue. When we say things like “You're so brave for trying this on your own,” we call this praising approach behaviors [sic]. We have this worksheet to help remind you of helpful things to say and what to avoid. As we do this practice exercise, Alex's mother please pay attention to when you want to say, “It's going to be ok.” This is usually a good time to say something encouraging - just try and [sic] praise approach behaviors instead (see Supplementary material for Worksheets # 1, #2, and #3).

### General ideas for the first exposure session

#### Relaxation strategies and coping skills

It should be noted that we do not emphasize teaching coping skills or relaxation strategies before starting the exposure sessions or using them while doing the exposures. This is because the research shows that they don't add much to treatment (38). Additionally, anything that is expected to “control” or “reduce” our experience of unpleasant emotions may actually do more harm than good (39). Even when it is not a compulsion, people can have a difficult time with relaxation strategies finding that they are “not working fast enough” and wondering if I am “doing it right.”

#### The role of mindfulness

Instead of relaxation or prior teaching of coping skills, we teach mindfulness skills *during* the exposure tasks. The key difference here is that, in mindfulness, we are trying to notice and non-judgmentally observe our thoughts, emotions, and physical sensations. We are *not* trying to change them in any way. We might notice our breathing but not do a whole breathing exercise. We might notice our muscle tension but not start progressive muscle relaxation. With multiple repetitions of a practice exercise, many of these things will change for the better. We want to notice what is happening, even though we aren't doing anything other than remaining present in the distressing situation. It is extraordinary that our body does this on its own and we don't have to micromanage it. In the context of ASD, individuals have difficulty practicing these skills out of context and knowing when to use them in context. Instead, we recommend diving into some exposures and teaching as one proceeds. It can sometimes be difficult to tell if we are teaching mindfulness or getting too close to relaxation strategies; for an in-depth comparison of the two [please see Luberto et al. (40)].

#### The example of Alex

In the remainder of this session, Alex repeated the exposure from the hierarchy of touching a sticky substance. We were able to build upon the last exposure by varying the stimuli from syrup to a few types of candies and we were able to extend the duration of the practice exercise to 20 min before washing. Alex's mother and the therapist both touched the sticky substances and the therapist asked questions designed to increase awareness of internal sensations and promote mindful engagement in the task (see session outline for more detail). The therapist prompted Alex's mother to use three or more of the encouragement statements during the task.

### Sessions four+: ongoing exposure sessions

All the sessions are scheduled for 60 min, or up to 90 min based on the clinic's setting. In general, ASD/OCD cases are at high risk for running over the allotted time. While it is agreeable to let the person leave when they are still distressed, we still try to be patient so that they are not leaving at a 9 or 10 out of 10. When planning these sessions, one needs to be mindful of their schedule so that it does not result in any additional time pressures. Scheduling these sessions before lunch or administration hour or at the end of the day tends to work well.

### Session outline

1. Review (up to 10 min): Review of homework and discussion of current stressors. Look for opportunities to praise approach behaviors. Give enthusiastic reflections and praise for any report of the patient identifying obsessions independently or even taking on additional challenges by reducing, delaying, or eliminating compulsions.
2. Core of the session (30–40 min): There is significant evidence that more exposure time means more treatment response. Plan exposures that are challenging but not overwhelming. Maximize the amount of time spent in exposure tasks and vary the stimuli to the amount that is tolerable.
3. End of session (10–15 min): Process the exposure with a focus on inhibitory learning (teaching the brain new ways to be mindful of distress without avoiding anxiety or trying to make it go away) and praise for approach behaviors. Assign homework to be completed by the following session.

### General ideas for continuing exposure sessions

#### Maximizing time in exposures

Sometimes patients chat as a conscious or subconscious attempt to reduce the time available for exposure tasks. When that happens, redirect and prompt them to engage in the exposure. At the start of treatment, this often means relying on forced choices. When we plan our sessions, we have to make sure to have stimuli prepared to complete three items from the patient's hierarchy at each session. We allow the patient to choose any of these three or something else. It is important to allow the “something else” option, as patients get further into treatment when they bring in their own challenge ideas. We want to reinforce these as this is an essential part of them incorporating ERP into their everyday life and setting the stage for maintenance of treatment gains after discharge.

#### Keeping the patient present

When in exposure tasks, prompt the patient to stay in the present moment and engage with the task. Ask them questions like, “What does the syrup feel like?,” “Is the dishwater hot or cold?,” “What does the ham taste like?,” and “What does the terrarium smell like?” By prompting the patient to notice small details and use all five senses, we keep them cognitively engaged with the task and limit internal avoidance, which maximizes the body's ability to eventually start habituation to the sensations with repeated practice and limits the likelihood of the anxiety becoming overwhelming during the session.

Additionally, it is important to continually prompt for discussion of internal sensations (somatic symptoms of anxiety) and passive noticing of thoughts (obsessions). For instance, a SUDS rating could be established while using a visual prompt, such as a thermometer or number line. Other conversational questions could be “What do you feel in your body?,” “What lets you know you're at a seven?,” “How do you know it changed from a seven to a six?,” “What is your brain saying?,” and “What is your OCD saying?” In the first several sessions, it is likely that individuals with ASD/OCD are unable to answer these questions. They may say, “I don't know,” or just not respond. They can be helped to develop these skills by giving them neutral observations like, “It looks like you're breathing a little heavier now,” “I can see you're holding tension in your shoulders,” “You're holding your arms stiff,” or “You look more relaxed because you're slouching on the couch.” This in-the-moment practice of internal awareness is helpful for individuals with OCD/ASD as they are often unable to think about these thoughts and feelings abstractly outside of the experience.

#### Processing the exposure

Processing questions capitalize on ideas of inhibitory learning by asking questions like: “Did the actual worst-case scenario happen?”, “Even if this was difficult, was it as impossible as OCD made it seem?”, “How long had you spent avoiding and dreading this task vs. how long did it take to complete it?”, “How long did it take for your body to adjust to this situation?”, and “Were you as inept in this situation as you thought?”. At the start of treatment, individuals with ASD/OCD often do not have much to say in response to these questions. It is important that the therapist provides these observations and praises their approach behaviors. It is important to continue asking these questions after each session, and as the treatment progresses, the patient will often build insight and start to be able to answer them.

#### Assigning homework

Homework assignments should be the same level of difficulty or slightly easier than the ones completed in the session. Ideally, they are the same as the task completed in the session but with different stimuli. For instance, if the patient was asked to cook a breakfast sandwich in the clinic, they could cook one at home. If they used a public bathroom in the clinic, they could use one at the grocery store. Making sure to vary the stimuli is important for the generalization of the skills to novel environments and situations. Socratic questioning, or asking a series of focused, open-ended questions that encourage reflection (41) should be used to help the patient generate as much of the homework assignment as possible. Over the course of treatment, the therapist's level of prompting can be faded out until the patient is generating assignments independently. For patients who have continually poor homework compliance, it must be considered if the assignments are too difficult or not defined specifically enough. For some tasks like emailing a teacher, the consequence of not completing the assignment at home could be that it was completed during the session.

#### The case of Alex

Alex needed about 40 sessions to meet our treatment responder criteria of 50% symptom reduction from the greatest measurement. Overall, Alex was cooperative with the exposure sessions and completed about 80% of the homework assignments. Most sessions followed the outlines above. There were several critical incidents that are worth discussing.

### Critical incidents

#### Alex's mother's therapy referral

During the third exposure session, Alex chose the practice task of washing the dishes in our therapeutic kitchen (our clinic has a kitchenette that we use for exposure therapy that mimics a home kitchen but also protects patient confidentiality by being separate from the general breakroom). It became clear during the task that Alex's mother was uneasy with this exposure. She was unable to use the encouragement statements she had used in previous sessions, appeared flushed, had changes in her breathing, and was less responsive to prompts. When asked how she was feeling, she said, “Maybe I have some of my own OCD.” The therapist finished the exposure task as previously defined with Alex and used the processing/homework time to discuss what was going on with Alex's mother was given the option of speaking to the therapist alone or with Alex present; she opted to have Alex present. She disclosed that she had her own history of anxiety that partially responded to a selective serotonin reuptake inhibitor (SSRI) and “talk therapy,” but after learning more and more about OCD, she thought she may have OCD. She accepted a referral to see another clinician in the same university-based clinic. She was eventually diagnosed with OCD herself and completed her own round of ERP. Her involvement in contamination or “just right” exposures was limited while she pursued her own treatment, but she continued to be present in practice tasks that were not personally triggering for her.

#### Locked in the bathroom

During the 10th exposure session, the therapist went to get Alex from the waiting room but did not see them. The therapist looked around and saw Alex's mother anxiously standing outside the bathroom door and trying to talk through the door. The therapist walked over, and Alex's mother indicated that Alex had become overwhelmed in the waiting room and had locked themselves in the bathroom. They were refusing to unlock the door, but stated they were safe. Alex's mother and the therapist sat down outside the bathroom door. Following is the transcript of the conversation between the therapist and Alex's mother with Alex listening in, until Alex joins in eventually.

*Therapist*: Tell me what happened before I came out here.
*Alex's mother:* Well, Alex wasn't thrilled about coming to session today. They said the sessions were getting hard and they were too tired. I did what you told me. I said I was proud of them for trying so hard and for keeping [sic] with it. They agreed to come but just looked so anxious. When we got here, we were a little early, so we sat to watch HGTV like usual. Then about 2 min before you came out, they just ran over here and locked the door.
*Therapist*: That all makes sense. Alex has been working hard in therapy and sometimes our OCD and our bodies get wound up when we think about the next challenge ahead.
*Alex's mother:* I know the therapy has been good for us. Alex is doing more around the house. It's been so nice to have them join us for movies and things. It's been nice that they can fix some of their own meals now. It's been good for me too. I've been feeling better about the dishes and other things.
*Therapist*: I agree. I've noticed improvements too. The fact that Alex is voluntarily in the bathroom is an improvement. (Mom laughs). It's ok, this is hard today; it doesn't mean that we are doing anything wrong. We're just going to sit here and be patient while Alex's body gets a chance to adjust.
*Alex's mother and the therapist continue small talk so that Alex could hear the therapist's voice and was unable to avoid their presence. After 5 min..*.
*Therapist*: I hope Alex can notice their breathing. Notice if it's speeding up or slowing down. Notice if they are breathing all the way into their belly or if it's getting stuck in their chest. Notice how their chest and back feel when they take a big breath. When Alex is ready, they could unlock the door. They don't have to open it yet, just unlock it.
*The bathroom door unlocks*.
*Therapist*: That's wonderful Alex. Good job approaching the situation. Your OCD is probably upset right now, but you're making such good choices to help yourself. No rush. We're going to be patient. Try and keep noticing your breathing. You can also notice what it feels like to be sitting on the floor, or the toilet, or wherever you are. Notice what it feels like to be supported. Notice if you feel heavy or where your muscles are tight. Are you tense in your back, neck, or face?
*Alex's mother:* I'm so happy you are here with us right now. I just don't know what to do in situations like this.
*Therapist*: Well, you're doing a good job keeping yourself outwardly calm. You might be upset on the inside, but it's helpful when you look calm on the outside so Alex can see things aren't out of control. Why don't you use some of your encouragement statements?
*Alex's mother*: Alex, sweetie, I'm so proud of you for getting in the car and getting here today. I know you were so anxious about coming here.
*Therapist*: That's great. Alex, when you're ready, can you open the door enough so I can slide my leg in and prop it open a bit? That way we can see each other.
*The door opens slightly. The therapist props the leg in. Alex is sitting on the floor with their back against the wall*.
*Therapist*: This is great. You're doing such a great job letting your body do whatever it's going to do and not running away from this situation. We're all doing a great job being patient.

Alex's mother and the therapist continue their small talk conversation, and Alex sits with their eyes closed, taking some deep breaths. After 10 minutes, Alex's shoulders relax down from their ears and their breathing looks normal.

*Therapist:* Alex, do you think you're ready to come to my office?

Alex nods yes and stands up slowly.

*Therapist*: Ok great. Let's go.
*Everyone goes to the therapist's office*.
*Therapist*: Alex, let's take a few minutes to adjust to the new room. I'm so happy you were able to be patient with yourself and get into my office. It's been a hard day already, but you're getting so much stronger than your OCD. You can keep noticing your breathing and again you can notice the couch, notice your muscles, notice your thoughts.
*Alex's mother and the therapist continue their conversation for 5 more min*.
*Therapist*: Do you think we are ready to practice something? I know it seems odd for me to ask after you basically just had a big practice exercise getting here, but we want to show OCD who's boss. We don't want OCD to learn that having an anxiety attack in the bathroom means no practice for the day. We can take it a little easier by completing the same practice we did last week.
*Alex*: Ok. We can do that. I don't want to, but I think I can do it.

The session ran over time by about 30 min. While this is often a challenge for clinic flow, this was ideal for this patient. They were able to complete the exposure task we initially set out to do (making a breakfast sandwich) and were able to learn how to manage anxiety attacks. The homework assignment was a repeat of the previous week. Making a breakfast sandwich at home was not included as it had not been included as a homework assignment yet and this would likely be too difficult to approach independently still.

The next five sessions had the same lead-up. After each session, the amount of time spent in the bathroom decreased as did the amount of prompting the therapist had to do. Alex continued to move up the hierarchy and complete assigned homework, but they did so a bit more slowly than they would have without these anxiety attacks. Going slower in this context meant allowing more repetition of assignments before changing the stimuli or adding a layer.

While the anxiety attacks were uncomfortable for everyone, including the therapist, we did not consider them a result of any therapeutic mistake. They ended up being a naturally occurring exposure for Alex and Alex's mother. They both were able to learn how to push through these uncomfortable moments to achieve their goals.

### Headbanging

In the 20th exposure session, Alex became distressed when trying to write an email to their teacher about a question on their homework. Alex was seated on the couch in the office with their feet up and their laptop on their lap. After some Socratic questioning about what to write to the teacher, Alex started banging their head on the soft, upper part of the couch. As the mild force of this behavior was deemed unlikely to cause damage, active ignoring was employed. The therapist stopped asking questions but did not remark or intervene in this behavior. After Alex stopped, they discussed with the therapist how this behavior was not entirely safe and would look odd to other people. They identified alternate behavior that could be used in its place and asked for a preferred fidget toy. They were able to resume the task with no additional headbanging. While it would have been ideal for Alex to write the email with no fidget toy, this accommodation was less destructive and more socially appropriate than the headbanging, so it was judged to be an appropriate allowance. Continued practice fading out the fidget toy was recommended in future sessions.

### Starting gender-affirming hormone therapy

During CBT-ERP, Alex was also being provided services at the university's gender clinic. The family was responsive to the psychoeducation provided. They started making changes to how they discussed Alex's gender, and they attended a local LGBTQ+ Pride event. They also resumed attendance at extended family gatherings and advocated for extended family members to respect Alex's identity. The largest change in mood during the entire course of treatment was when Alex started testosterone. They came to that session smiling unlike any session prior and reported feeling excited about the medication. Alex's depressive symptoms decreased to subclinical levels shortly after starting testosterone and remained low for the rest of the treatment.

#### Progress monitoring

**Figure 5:**
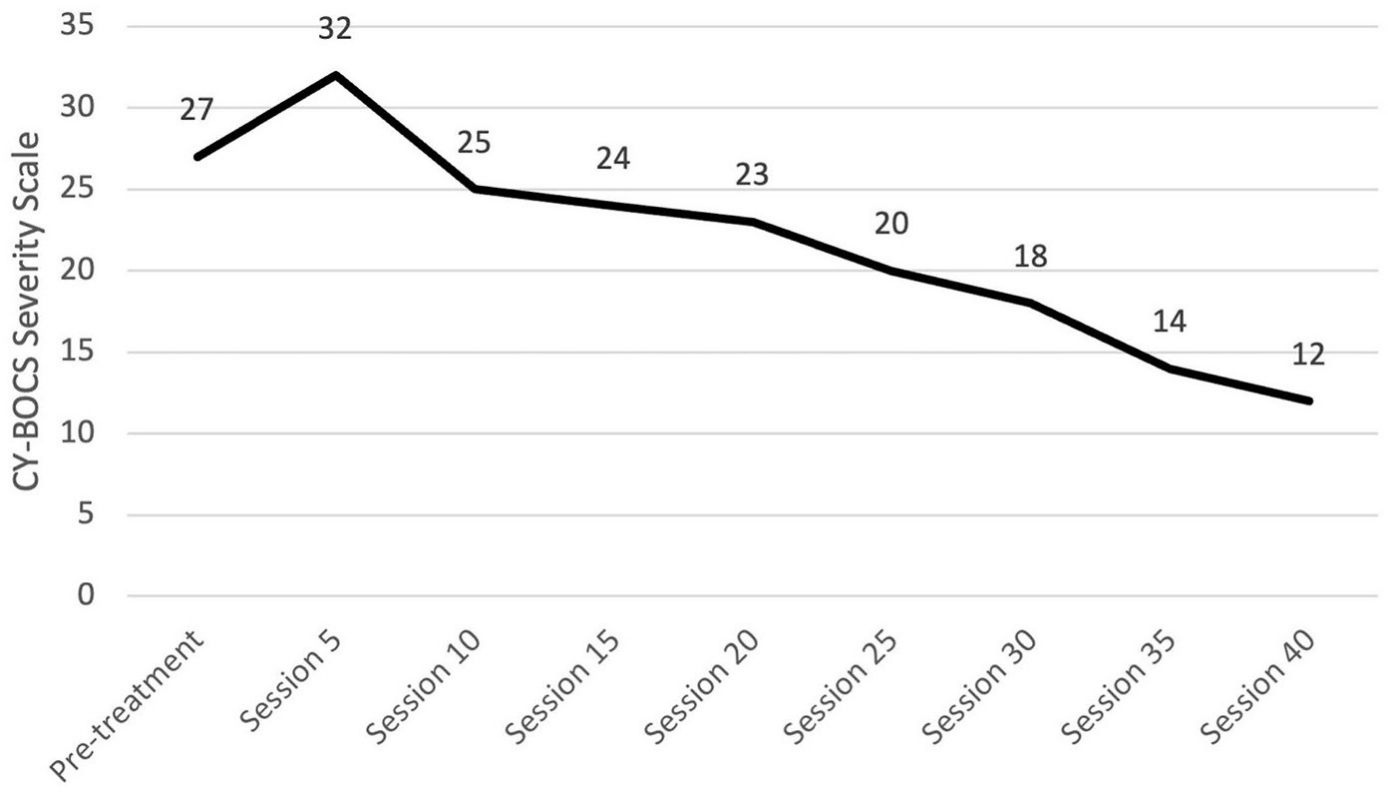
Change in OCD symptom severity throughout treatment. OCD, Obsessive-compulsive disorder; CY-BOCS, Children's Yale-Brown Obsessive-Compulsive Scale.

**Figure 6:**
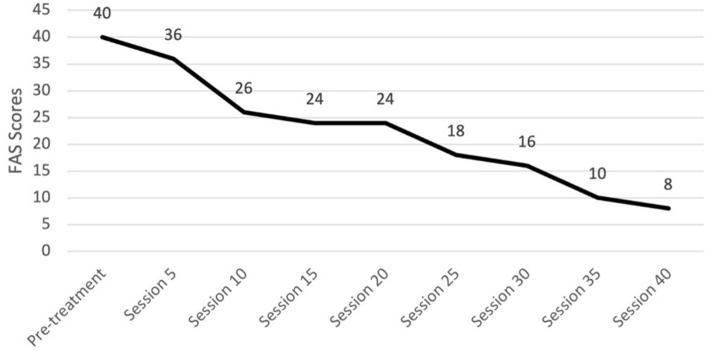
Changes in family accommodation throughout treatment. FAS, Family Accommodation Scale.

We recommend the use of the CY-BOCS severity scale and FAS every five sessions. We preferred a clinician-administered CY-BOCS since it allows the clinician to integrate information from both the patient and family (23). One should not be disheartened if there is an initial worsening of the CY-BOCS from intake to the fifth session; this commonly occurs as the patient and their family build insight into what is truly attributable to OCD. The CY-BOCS score will start to decline as one progresses through the tenth session, though it may be a slow process. Alex's CY-BOCS scores were higher at session five than at pre-treatment but then decreased gradually over the course of treatment, as expected (see Figure 5). The FAS could be used to set homework goals for the patient and family that focus on limiting accommodation. Alex's mother's self-reported FAS total scores steadily decreased across sessions (see Figure 6).

**Figure 7:**
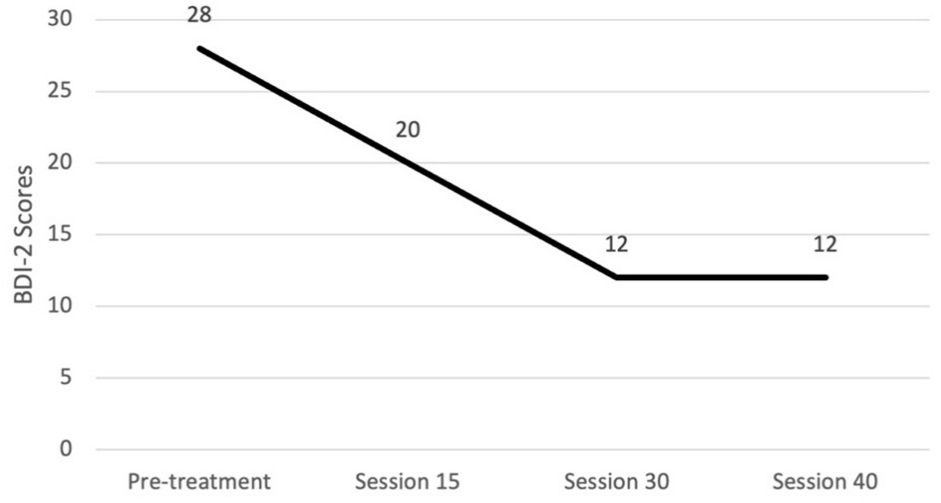
Changes in depression severity throughout treatment. BDI-2, Beck Depression Inventory - Second Edition.

As discussed, OCD is highly concurrent with other disorders. We recommend tracking co-occurring symptoms at regular intervals. For Alex, we decided to also track their depressive symptoms since they were elevated and part of the gender dysphoria diagnosis. Alex's depressive symptoms were assessed using the BDI-2, and these scores also decreased over the course of treatment. Graphs summarizing Alex's change in CY-BOCS, FAS, and BDI-2 can be found in Figures 5–7, respectively.

### Follow-up and outcomes

After approximately 40 exposure sessions, Alex's CY-BOCS score had decreased to the mild range, and symptom severity had decreased by more than 50% from its greatest measurement. Parent-reported FAS score indicated minimal accommodation. Alex's BDI-2 score was also in the minimal range. Functionally, they were using the bathrooms at home as usual, making many of their own meals, spending time with the family both at home and outside the home, and most notably, had returned to virtual schooling. In these classes, they were able to keep their camera on, ask questions, and complete assignments. Alex scheduled a 1-month follow-up appointment at which time targeted skills were still going well. The CY-BOCS increased slightly to a total score of 13 but remained in the mild range. Alex's mother was anxious about terminating treatment; therefore, one more 1-month follow-up was scheduled. At that time, progress was maintained, and the CY-BOCS total score was 11. Qualitatively, functional gains had been maintained and family accommodation and conflict around OCD symptoms remained low. The following follow-up care was recommended.

1. Given that individuals with ASD and gender concerns tend to have ongoing social adjustment issues throughout adolescence and early adulthood, it was recommended that they establish care with a community provider who would be able to follow them long-term.
2. It was recommended that they follow up with their endocrinologist at the Gender Clinic regarding the ongoing use of testosterone and any other medical interventions.
3. Continued consultation with a psychiatrist regarding medication management of both ADHD and OCD was encouraged. During treatment, Alex did add an SSRI to their medication regime and did find it helpful for the OCD.

## Discussion

There were several strengths in our treatment approach. First, we were able to identify the common characteristics of individuals who have ASD and were assigned female at birth and properly evaluate for both ASD and OCD. The ability of our clinicians to provide ERP and to complete comprehensive ASD evaluations made the referral process nearly seamless for the patient. In some clinics, these services are provided by separate clinicians leading to increased time on waitlists, subsequent delays in starting treatment, and poor communication between providers. Second, we believe that properly identifying and treating the mother's OCD was also a strength. Without the intervention at the level of the mother, it was unlikely that the patient would progress through treatment or maintain treatment gains.

Next, we were fortunate to have a gender clinic as part of our university health system. By being able to place this referral, we were able to focus our efforts on the OCD treatment. Without this resource, time would have had to be split to address the family dynamics around gender as these ongoing conflicts would make engagement in ERP challenging. From the provider's perspective, the greatest weakness of our treatment modality was the need to refer out for follow-up care. As a specialty treatment center, we were unable to keep patients indefinitely, and instead, we followed an episode of care model so that, after measurable treatment gains have been obtained, the patient is referred out. Unfortunately, community providers with knowledge of OCD, not to mention the ASD/OCD overlap, are hard to come by and follow-up care may not always be as effective as we would like. Hopefully, continued dissemination of research and clinical applications will increase the number of providers able to provide such follow-up.

Most importantly, we believe that our integrated approach to ERP in the context of ASD is a contribution to providers who are new to either ASD or OCD evaluations and treatments. We believe our approach is well-founded in the literature and have worked hard to outline that foundation in the companion piece (1). However, this approach has not yet been studied enough to meet the criteria of an EBT. Therefore, additional research needs to be done before we can claim to have an evidence-based manual for ERP in the context of OCD.

## Conclusion

Providing high-quality, evidence-based care for youth with co-occurring ASD and OCD requires extensive background knowledge, clinical judgment, flexibility, and sensitivity. Practitioners must be competent in selecting and administering appropriate, psychometrically sound assessment measures, several of which are described in this article, for differential diagnosis among often overlapping symptoms (e.g., repetitive behavior, avoidance, and social impairment). Relatedly, it should be noted that careful attention must be paid to commonly co-occurring symptoms related to attention, anxiety, mood, repetitive behavior, and gender diversity. As the field's understanding of EBTs for OCD in adolescents with ASD emerges, providers should seek consultation and refer to the literature often. Best practices, measurement tools, and neurodevelopmental science are likely to change as further research is conducted.

## Patient perspective

Because this is a fictionalized case study, we could not solicit the patient's perspective directly. We can report qualitatively on common reflections from our patients. Patients often say that ERP was the “hardest thing” they ever did but that it “helped” and that they are happy they saw it through. Similarly, parents report that they found changing their behavior to be difficult and that they disliked seeing their child in distress but were happy about their gains in functioning and agree there is less distress overall now. The most common difficulty for patients that complete treatment is that they do not want to end their care. Especially in the context of ASD where continued therapy is indicated for ongoing adjustment concerns in the adolescent developmental period, patients do not want to “start over” with a new provider and are worried that they will not be able to access care soon enough if symptoms return. For these fears, we approach them like any other and attempt not to accommodate them past two 1-month follow-up sessions.

## Data availability statement

The original contributions presented in the study are included in the article/Supplementary material, further inquiries can be directed to the corresponding author.

## Author contributions

AG conducted clinical cases, conceptualized the manuscript, wrote manuscript sections, and revised the manuscript. CL conceptualized the manuscript, wrote manuscript sections, and revised the manuscript. BB conducted clinical cases, conceptualized the manuscript, wrote manuscript sections, and edited the manuscript. MM and MB-H conducted clinical cases, wrote manuscript sections, and edited the manuscript. MH and LD wrote manuscript sections and edited the manuscript. CM conceptualized and revised the manuscript. All authors contributed to the article and approved the submitted version.
