# Supplementary material for: Case report: Co-occurring autism spectrum disorder (Level One) and obsessive-compulsive disorder in a gender-diverse adolescent
Source: Front Psychiatry. 2023 May 16;14:1072645. doi: 10.3389/fpsyt.2023.1072645 (PMC10227521; doi:10.3389/fpsyt.2023.1072645)
Supplement: Supplementary file 1 [file Table_1.docx]

**How to Create Great Labeled Praises for Approach Behavior**

| That’s a great way to… | face your fears. |
| --- | --- |
| You’re doing a nice job of… | challenging OCD. |
| I like it when you… | talk back to OCD. |
| It’s neat that you remembered to… | do your homework on your own. |
| What a wonderful idea to… | try something new. |
| Thank you for… | being patient. |
| Nice job of… | staying focus on the task. |
| How sweet of you to … | notice I’m trying to help. |
| You should be proud of yourself for… | completing a difficult exercise today. |
| I’m proud of you for… | showing up even though you were tired. |
| You are so smart to… | use your ERP skills. |
| I’m so happy with you for… | challenging yourself. |
| Super… | job staying present in this moment. |
| Good… | job sticking with it. |
| I like it when you… | surprise yourself. |
| It’s cool that you are… | finding your own ways to challenge yourself. |
