# Supplementary material for: Case report: Co-occurring autism spectrum disorder (Level One) and obsessive-compulsive disorder in a gender-diverse adolescent
Source: Front Psychiatry. 2023 May 16;14:1072645. doi: 10.3389/fpsyt.2023.1072645 (PMC10227521; doi:10.3389/fpsyt.2023.1072645)
Supplement: Supplementary file 2 [file Table_2.docx]

**How to Replace Reassurance Statements**

| **Instead of…** | **Say…** |
| --- | --- |
| It’s going to be ok. | I know you can handle it. |
| Everything is fine. | You’re being so brave. |
| Don’t worry. | Your OCD is loud right now. |
| You don’t have to do this. | I’m so proud of you for sticking with it. |
| I would never let anything bad happen. | I believe in you. |
| You can’t get sick from this. | Who knows? People get sick all the time. |
| You aren’t going to stab me. | Anything is possible. |
| I know that wouldn’t happen. | I don’t know what’s going to happen. |
| I know you wouldn’t do that. | Let’s see what happens. |
| This is too hard for you. | You’re doing great challenging yourself. |
| Just let me do it. | I can wait while you do this for yourself. |
| That’s too gross. That’s impossible. | Wow! I’m so impressed. That’s even hard for me to do. |
