# Supplementary material for: Case report: Co-occurring autism spectrum disorder (Level One) and obsessive-compulsive disorder in a gender-diverse adolescent
Source: Front Psychiatry. 2023 May 16;14:1072645. doi: 10.3389/fpsyt.2023.1072645 (PMC10227521; doi:10.3389/fpsyt.2023.1072645)
Supplement: Supplementary file 3 [file Table_3.docx]

**Noticing Approach Over Avoidance/Compulsions**

| **Avoidant/Compulsive Behavior** | **Approach Behavior** | **Praise** |
| --- | --- | --- |
| Saying “I’ll do [it] later.” | Doing [it] right away. | Thanks for doing [that] so quickly. |
| Asking “are you sure?” | Accepting the first answer. | Great job hearing me the first time. |
| Using up the soap. | Saving some soap. | Thanks for leaving some soap for the dishes tonight. |
| Asking you to complete a ritual. |  |  |
| Saying “I don’t want to go.” |  |  |
| Asking for someone else to do a chore. |  |  |
| Lying about homework being due. |  |  |
| Being bossy. |  |  |
| Not allowing others in their space. |  |  |
| Showering routines that occupy the bathroom. |  |  |
| Moring routines that make you late to school. |  |  |
| Nighttime rituals that keep you up. |  |  |
